# Locust Bean Gum/κ-Carrageenan Film Containing Blueberry or Beetroot Extracts as Intelligent Films to Monitoring Hake (*Merluccius merluccius*) Freshness

**DOI:** 10.3390/foods13193088

**Published:** 2024-09-27

**Authors:** Carla S. V. Faria, Jorge M. Vieira, António A. Vicente, Joana T. Martins

**Affiliations:** 1 CEB—Centre of Biological Engineering, University of Minho, 4710-057 Braga, Portugaljorgevieira@ceb.uminho.pt (J.M.V.); avicente@deb.uminho.pt (A.A.V.); 2 LABBELS—Associate Laboratory, Braga/Guimarães, Portugal

**Keywords:** bio-based film, pH indicator, anthocyanins, betalains, shelf-life

## Abstract

The main goal of this work was to develop bio-based and ecofriendly intelligent films as freshness indicators to monitor European hake (*Merluccius merluccius*) quality during storage by using a visual, non-destructive, and real-time technique. Locust bean gum (LBG)/κ-carrageenan (Car) films incorporating blueberry extract (BLE) or beetroot extract (BEE) were developed and their effectiveness to detect hake deterioration during 7 days of storage at 4 °C was evaluated. A visible color response from pink to blue was observed on the BLE films at the end of hake storage, which correlated with the hake deterioration profile, namely an increase in pH values (from 6.60 ± 0.04 to 8.02 ± 0.03), total viable count (TVC, from 4.61 ± 0.36 to 8.61 ± 0.21 log CFU/g), and total volatile basic nitrogen content (TVB-N, from 10.21 ± 1.97 to 66.78 ± 4.81 mg/100 g) beyond the spoilage threshold. The results of this study are very promising, since it was possible to develop a new effective intelligent bio-based responsive indicator film incorporating natural dye BLE, which has the potential to contribute to food waste reduction and improve food safety by detecting the hake freshness status.

## 1. Introduction

Food products that exhibit safety limitations due to the presence of microbial agents (bacteria, viruses, and parasites) and/or harmful chemical substances are the cause of more than 200 diseases including diarrhea and cancer, according to the World Health Organization (WHO) [1]. Food deterioration as a result of biological, chemical, or physical phenomena causes changes, in some cases, not directly recognized by the final consumers. Moreover, approximately 30% of all food produced worldwide is lost or wasted every year along the supply chain, according to the Food and Agriculture Organization (FAO). Category wise, food loss is predicted to be 20% in dairy products, 35% in seafood, 45% in fruits and vegetables, and 20% in meat, with the losses predominant at the processing, distribution, and consumption stages [2]. Therefore, it is important to solve the current challenges of food waste, quality, and safety through (i) the detection of changes in food products, in order to avoid product consumption by the final consumer; (ii) the identification of potential health risks; and (iii) the establishment of strategies to reduce or eliminate the occurrence of the previously mentioned events [3].

In this context, the development of innovative packaging with specific functions, namely intelligent packaging, has been observed in recent years. Intelligent packaging emerged with the aim to detect, monitor, and inform the consumer about the quality of food throughout the food chain in a non-destructive way and in real-time [4,5]. Moreover, intelligent packaging has been an important contribution to avoid unnecessary food waste because it is able to monitor and display the food quality status, and consequently avoid food products being thrown away even though they are still suitable for consumption. On the other hand, it has the potential to improve product safety, food logistics, and traceability, increasing the food industries’ efficiency [4].

Freshness indicators (such as pH indicators) are devices usually integrated into the packaging that are capable of reacting with metabolites generated by microbial growth and metabolism (e.g., organic acids, ethanol, biogenic amines, and volatile nitrogen compounds) or are able to detect any chemical change, providing visual information about the food product quality [6]. As synthetic dyes are considered carcinogenic or mutagenic agents in humans, their application in food intelligent packaging is not ideal. In an attempt to overcome this limitation, more attention has recently been given to the alternative use of natural pigments. Natural dyes (pigments) extracted from plants are attractive and relevant options in the intelligent packaging field due to their low toxicity, easy preparation, renewable, and non-polluting properties [1,7,8]. Examples of natural dyes that change their color significantly with pH changes are anthocyanins and betalains [9,10,11].

The increase in consumer and food industry consciousness related to food safety and environmental pollution has guided researchers to focus on the development of safe, environmentally friendly, and biodegradable packaging materials. Thus, our research aim was to replace synthetic, petroleum-based polymers (such as plastics) with eco-friendly materials to be used in food packaging, especially in intelligent packaging [5,12]. The interest in using natural biopolymers such as polysaccharides for the development of bio-based intelligent packaging has increased due to their high abundance and high availability in nature and in low-value agricultural residues/by-products (for example, fruit peels and seeds) [5,10]. For instance, intelligent packaging films have been developed based on locust bean gum (LBG) and polyvinyl alcohol containing betacyanins from the cockscomb flower to indicate shrimp freshness [13]. κ-Carrageenan (Car) is an anionic linear sulfated polysaccharide that consists of the repetition of disaccharide units with 3,6-anhydro-D-galactopyranose residues in its chain [14]. These have been widely used in the food (mainly in dairy products) and pharmaceutical industries due to their biocompatible and biodegradable properties. He et al. [15] developed a film based on Car, gelatin, and curcumin, which work as an intelligent indicator for the freshness detection of grass carp fillets. Thus, polysaccharide-based intelligent films with natural compounds as freshness indicators have the potential to guarantee food quality and safety, reduce environmental impact, and increase the product packaged attractiveness to the retailer and the final consumer [7,12]. However, despite permanent innovations in the food packaging area, research related to this type of bio-based intelligent packaging is still limited.

Fish consumption and popularity have been increasing due to the customers’ awareness regarding fish nutritional composition, especially its high protein content [16]. Several studies have been carried out to monitor the freshness of fish products such as Atlantic mackerel (*Scomber scombrus*) and Wuchang bream (*Megalobrama amblycephala*) using bio-based intelligent films containing natural pH dyes [11,17]. However, there is a lack of information regarding hake quality monitoring using bio-based intelligent packaging, which was a research opportunity explored in the present work. Portugal is the largest fish consumer per capita in the European Union due to its geographical location and tradition in fishing and fish consumption, with hake (*Merluccius merluccius*) the third most consumed fish in Portugal [18,19]. Hake is highly perishable due to microbial contamination and enzymatic reactions and can cause high economic losses for the fishery industry as well as a high risk to human health, thus it is crucial to monitor the fish freshness during storage, processing, and transportation.

Thus, to tackle the current concerns related to the consumption of contaminated fish products, food waste, and the environmental impact of petroleum-based packaging, a novel quality indicator sensitive to the changes in hake fish freshness was developed in this work. Therefore, a colorimetric bio-based LBG/Car intelligent film incorporating natural dyes—blueberry extract (BLE) or beetroot extract (BEE)—was developed. Moreover, the effect of BLE or BEE on the LBG/Car films’ mechanical, water barrier, and optical properties as well as color response efficiency to different pH conditions and volatile nitrogen compounds were assessed.

## 2. Materials and Methods

### 2.1. Materials

Polysaccharides, Car (Gelcarin^®^ DG 5264), and LBG were obtained by FMC Biopolymer (Philadelphia, PA, USA) and Sigma-Aldrich (St. Louis, MO, USA), respectively. The glycerol was purchased from Sigma-Aldrich (St. Louis, MO, USA). Freeze-dried BLE powder (Simplu^®^, Guimarães, Portugal) and BEE powder (Solgar^®^, Leonia, NJ, USA) were purchased in Celeiro (Braga, Portugal). Sodium hydroxide (NaOH), ammonia, and plate count agar (PCA) were provided by Merck (Merck SA, Lisbon, Portugal). Boric acid (H_3_BO_3_), hydrochloric acid (HCl), and sulfuric acid (H_2_SO_4_) were supplied by Chem-Lab (Chem Lab, Zedelgem, Belgium). Potassium carbonate (K_2_CO_3_) and methyl red indicator were purchased from Panreac (Panreac AppliChem ITW Companies), Barcelona, Spain). Bromocresol green was supplied by Acros Organic (Acros OrganicsTM, Lisbon, Portugal). The fresh hake was purchased from a local market (Braga, Portugal).

### 2.2. Production of LBG/Car Film Incorporating BLE or BEE

LBG/Car films were produced based on our previous works [20,21]. Initially, 60% (*w*/*w*) LBG and 40% (*w*/*w*) Car were dissolved in distilled water under stirring for 1 h at 25 °C. Then, 30% (*w*/*w*) of glycerol was added, and the film-forming solution was homogenized at 70 °C under agitation for 30 min. This film-forming solution was used to produce control films (i.e., no-added BLE or BEE) and films incorporating the extracts (1 to 50% (*w*/*w*)). Regarding films incorporating extracts, 30% (*w*/*w*) BLE or 50% (*w*/*w*) BEE was added to the LBG/Car film-forming solution and homogenized at 1200 rpm for 30 min at 40 °C. The selected BLE and BEE concentrations were based on preliminary tests, where the films’ visual color uniformity and handling behavior (e.g., brittle) were assessed. All film-forming solutions were added to Petri dishes (90 mm) and placed in an oven with airflow circulation (WTC Binder) at 35 °C for 22 h to produce the films. All films produced were conditioned in a desiccator at 54% relative humidity (RH) until further analysis.

### 2.3. Physico-Chemical Properties of BLE and BEE Films

#### 2.3.1. Color and Opacity of the Films

The color of films was determined with a Minolta colorimeter (CR 300; Minolta, Japan) [21,22]. To calibrate the instruments, a white standard color plate (*Y* = 93.90; *x* = 0.32; *y* = 0.33) was used as the background for the film color measurements. Lightness (L*) (white to black) and chromaticity parameters a* (red to green) and b* (yellow to blue) were assessed through reflectance measurements using the CIELab scale. The color change of films during storage (Δ*E*) was determined using Equation (1):

(1) $$∆E=\sqrt{\left[{(∆L*)}^{2}+{(∆a*)}^{2}+{(∆b*)}^{2}\right]}$$

The opacity of each film sample was obtained according to the Hunterlab method as the relationship between the film opacity on a black standard plate and on a white standard plate. All color and opacity measurements were performed six times for each sample, and three samples of each film were tested.

#### 2.3.2. Evaluation of Color Stability of the Films during Storage

The BLE and BEE films were stored under light or dark conditions for 60 days at 25 °C or 4 °C to evaluate their color stability during storage. The L*, a*, and b* colorimetric parameters were measured in each film using a CR400 colorimeter (Konica, Minolta, Osaka, Japan) according to the methods described in the literature [8,12]. The color parameters of the films were analyzed at 2, 5, 10, 15, and 60 days of storage. The colorimetric measurements were conducted in triplicate on each film sample.

#### 2.3.3. Evaluation of pH-Sensitive Properties of the Films

In order to evaluate the BLE or BEE films’ color response to pH changes, a droplet of the HCl (0.1 mol/L) aqueous solution (pH 2, 4, and 6), distilled water (pH 7), or NaOH (0.1 mol/L) aqueous solution (pH 8, 10, 12 and 14) was placed on the surface of the film samples (2 × 2 cm) for 10 min [10]. The color change was evaluated by comparing the color differences between the films before and after contact with the buffer solutions. All measurements were taken at three random locations of each film sample.

#### 2.3.4. BLE and BEE Films Response to Ammonia Vapor

The film’s color response to volatile compounds, namely ammonia vapor, was determined as described by Qin et al. [23] and Yun et al. [24]. BLE and BEE film samples (1.5 × 1.5 cm) were placed in the headspace of sealed Petri dishes loaded with 0.4 mol L^−1^ ammonia solution for 60 min at room temperature under no-light conditions. The L*, a*, and b* colorimetric parameters of the films were measured with 10-minute intervals. Color measurements were conducted at three random locations of each film sample.

#### 2.3.5. Water Contact Angle (WCA) Measurements

The WCA on the film surface was measured using an optical contact angle meter (OCA 20, Dataphysics, Filderstadt, Germany) through the sensile drop method [25]. Each film sample was cut into square pieces (1.5 × 1.5 cm) and 5 μL of ultrapure water was dropped onto the film surface using a micro-syringe with a 0.75 mm diameter needle (Hamilton, Bonaduz, Switzerland). Contact angle measurements were performed after 30 s of the droplet placement on the film surface.

#### 2.3.6. Moisture Content (MC) and Water Solubility (WS)

The MC and WS of the films were determined gravimetrically according to the methods reported by other authors [22,26]. The MC was determined by measuring the weight loss of the film samples (2 cm of diameter) after drying at 105 °C in an oven (WTC Binder) until it reached a constant dry sample weight. To determine the WS, dried samples of the film (2 cm of diameter) were weighed and immersed in 50 mL of deionized water for 8 h at room temperature under orbital agitation at 120 rpm (Orbital shaker ES-20/80, BOECO, Hamburg, Germany). The water was removed and the film residue was dried at 105 °C for 24 h to determine the weight of dry matter that was not solubilized in water. For the MC and WC assays, three replicates were performed for each film sample.

#### 2.3.7. Water Vapor Permeability (WVP)

The WVP of the films was determined gravimetrically following the methodology developed by other authors [22,25]. Three samples were cut from each film and sealed on a permeation cup cell containing 50 mL of distilled water (100% RH; 2337 Pa vapor pressure at 20 °C) and placed in a desiccator containing silica gel (0% RH; 0 Pa water vapor pressure at 20 °C). Constant air circulation outside of the test cup was maintained by using a fan inside the desiccator to assure uniform water pressure and steady-state conditions. Cups were weighed every 2 h for 12 h, and the water transferred through the film was determined from the weight loss of the permeation cup cell. All measurements were performed in triplicate. The water vapor transmission rate (WVTR) through the film (g m^−2^ s^−1^) was calculated through the ratio between the slope of the linear regression of weight loss versus time and the film area. The WVP (g m^−1^ s^−1^ Pa^−1^) was calculated according to Equation (2):

(2) $$\mathrm{WVP}=\frac{\mathrm{WVTR}\;\times \;L}{∆P}$$

where L is the mean film thickness (m) and Δ*P* is the partial water vapor pressure difference (Pa) across the two sides of the film.

Film thickness was measured using a digital micrometer device (Mitutoyo No. 293-766, Kanagawa, Japan) with 0.001 mm accuracy. Ten thickness measurements were randomly taken from different points of each film sample [27].

#### 2.3.8. Assessment of Mechanical Properties

The tensile strength (TS) and elongation-at-break (EB) of the films were measured using a TA-HD plus Texture Analyzer (Serial RS232, Stable Micro Systems, Surrey, UK), according to Martins et al. [21]. Film specimens (120 mm × 20 mm) from each film sample were cut and placed between the tensile grips. The initial grip separation and crosshead speed were set at 30 mm and at 5 mm min^−1^, respectively. The TS and EB were determined from the stress–strain curves. At least four measurements were performed for each film.

### 2.4. Evaluation of BLE and BEE Films as Hake Freshness Colorimetric Indicators

#### 2.4.1. Sample Preparation and Storage Conditions

Fresh hake was purchased from a local market (Continente, Braga, Portugal) and immediately transported to the laboratory. Fishbones were removed, and fish fillet samples (approximately 5 g) were prepared.

**Figure 1:**
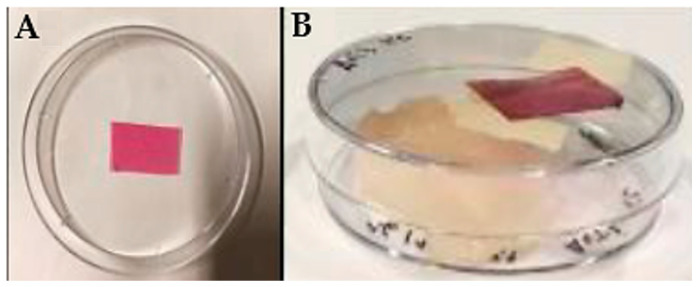
Illustrative example of the experimental setup used to monitor hake freshness during storage at 4 and 25 °C. (**A**) Film sample fixed to the Petri dish cover and (**B**) closed Petri dish system containing the hake sample used to monitor the freshness status.

To validate the BLE and BEE films as colorimetric indicators sensitive to pH and gas changes, tests with fish samples stored at 4 °C and 25 °C for 7 days were carried out. BLE and BEE film samples (2 cm × 2 cm) were fixed to the top cover of sterile polystyrene 45 mm diameter Petri dishes that were in contact with the dish headspace, as can be seen in Figure 1. Then, the fish samples were placed into Petri dishes and sealed [12,26] (Figure 1). The Petri dish with no-added fish sample was used as the control. All samples were stored at 4 °C and 25 °C and analyzed on days 0, 2, 3, 4, 6, and 7 to detect any physico-chemical or microbial changes. All results obtained over the storage period were compared with the results obtained on day 0. For each sampling day, three replicates were performed for each fish sample.

#### 2.4.2. Film Color Analysis

Film color changes during the loss of hake freshness over the storage time were assessed according to the method described in Section 2.3.1. At least three color measurements were performed on different film samples on each sampling day.

#### 2.4.3. pH Measurement of Fish Samples

The fish samples were blended with 100 mL of distilled water using a stomacher (Stomacher^®^ 3500, Sweard Medical, Worthing, UK) at high speed for 4 min. All pH measurements were performed using a digital pH meter (HI 2210, Hanna Instruments, Porto, Portugal) by immersing the glass electrode in the homogenized samples [12].

#### 2.4.4. Determination of Total Volatile Basic Nitrogen (TVB-N)

TVB-N measurements were conducted according to the Conway micro diffusion method with some modifications [15]. Initially, the hake samples (4 g) were added to 40 mL of distilled water and homogenized using a stomacher (Stomacher^®^ 3500, Sweard Medical, UK) for 8 min. Then, the mixture was filtered using filter paper (type 601A Rotilabo^®^-Round filters, Carl Roth, Karlsruhe, Germany). Subsequently, 1 mL of 0.02% boric acid and one drop of pH indicator (methyl red:bromocresol green = 1:5) were added to the inner chamber of the Conway dish. One mL of saturated potassium carbonate solution and 1 mL of the fish filtrate sample were added to the outer dish chamber. A blank sample was also prepared as described, but the filtered sample was not added to the Conway dish. The lid of the Conway dish was closed, and the resulting mixture was allowed to stand at room temperature for 1.5 h. Finally, the solution in the inner chamber was titrated with hydrochloric acid (0.01 mol/L) until a pale pink color appeared. Analyses were conducted in triplicate. The TVB-N content (presented as mg N/100 g of fish sample) was calculated using Equation (3):

(3) $$TVB-N=\frac{\left(V1-V2\right)\times c\times 14\times 100}{m\times \frac{V}{V0}}$$

where V1 and V2 represent the HCl volume (mL) required for the sample and blank titration, respectively; c is the HCl concentration (mol/L); m is the fish sample weight (g); 14 is the molecular weight of nitrogen; 100 is the conversion factor; V is the volume of filtrate sample (mL); and V0 is the total volume (mL).

#### 2.4.5. Microbiological Analysis

The determination of the total viable count (TVC) was performed in triplicate according to the procedures reported by other authors [15,27]. Hake samples (4 g) were transferred to a stomacher bag. Then, 36 mL of sterile 0.1% peptone water was added to the bag and homogenized for 2 min using a stomacher. Serial decimal dilutions (10^1^ to 10^6^) were prepared, and 1 mL of the appropriate dilution was placed in a Petri dish and 20 mL of sterile PCA was added to the dish. The dishes were incubated at 35 °C for 72 h. The TVC was determined and expressed as a log colony forming unit per g fish sample (log CFU/g). According to the TVC results , the initial TVC of the hake samples was 4.56 ± 0.36 log CFU.g^−1^.

### 2.5. Statistical Analysis

All experiments were performed at least in triplicate. All data were expressed as the mean values ± standard deviation (SD). One-way analysis of variance (ANOVA) was performed, and the significant differences among samples were determined by the Tukey HSD test (*p* < 0.05). Statistics were performed on the SigmaPlot Software 11.0 for Windows.

## 3. Results and Discussion

### 3.1. Development of LBG/Car Film Incorporating BLE or BEE

**Figure 2:**
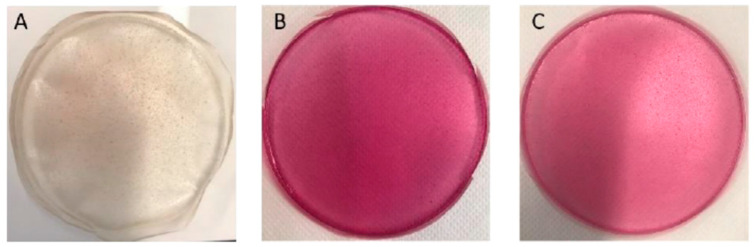
Photographs of the (**A**) control film (no-added extracts), (**B**) film incorporating 30% (*w*/*w*) BLE, and (**C**) film incorporating 50% (% *w*/*w*) BEE.

Different BLE or BEE concentrations were tested to select the films’ formulations for use as potential freshness colorimetric indicators. LBG/Car films presented a soft yellow color and were transparent, while films incorporating BLE or BEE showed a pink color (Figure 2). Based on visual examination of the film surface, namely the color uniformity throughout the film and handling properties, films containing 30% (*w*/*w*) of BLE or 50% (*w*/*w*) of BEE concentrations were selected for further analysis. Films containing BLE and BEE concentrations below 30% and 50%, respectively, presented very light and non-homogenous coloration and were very brittle when handled.

### 3.2. Physicochemical Properties of the Developed Films

#### 3.2.1. Optical Properties: Color and Opacity

The color parameters (L*, a*, and b*) and opacity of the various films developed are shown in Table 1. The control film showed a higher transparency and luminosity (i.e., higher L* value) than the BLE and BEE films. The BLE addition to the film led to a decrease in L* and b* parameters (*p* < 0.05), which demonstrated a decrease in film brightness and an increase in the blue color of the film (Table 1). On the other hand, there was a significant increase in a* value (*p* < 0.05) compared to the control film, which represented an increment in red coloration of the film. This result was in line with the reddish/purple color of the BLE, contributing to the red/purple color of the BLE film. Qin et al. [5] also concluded that the rise in the anthocyanin-rich extract from *Lycium ruthenicum* Murr content within starch films significantly increased the a* value and decreased the b* value, indicating a change in film color to red. Merz et al. [28] observed that the color of the chitosan and polyvinyl alcohol film changed to red when anthocyanins from the jambolana fruit were added. The addition of BEE also caused a decrease in the L* and b* values (*p* < 0.05) of the control films (Table 1). Furthermore, the a* value of the BEE film had a higher increase when compared to the BLE film (*p* < 0.05). For this reason, the pink/red color was more visible on the BEE films (Figure 2). These changes in the color parameters after adding red pigments such as betalains from beetroot have previously been reported. For instance, Wu et al. [13] showed that films based on LBG/polyvinyl alcohol with betacyanins from the cockscomb flower were reddish-purple, which caused an increase in the a* value. A similar color was also observed in pectin-based films incorporating the beetroot extract [29].

Film opacity refers to the film’s ability to act as a barrier to light. The higher the opacity value, the lower the film transparency. The control film showed the lowest opacity value compared to the BLE and BEE films, confirming its high transparency (Table 1). The BLE film presented the highest opacity value compared to the BEE films. Thus, the BLE film reduced the film transparency and hindered the passage of light through it, which has great potential to prevent oxidative rancidity in foods caused by exposure to light [30]. Other works have reported similar film opacity results where the increase in film opacity was due to the incorporation of natural extracts in bio-based intelligent films [31,32].

#### 3.2.2. Color Stability of the BLE and BEE Films during Storage

It is necessary to evaluate the influence of environment conditions (namely, temperature and light) on the produced films in order to confirm that the intelligent film can provide reliable visual feedback to consumers. Thus, the BLE and BEE films’ color stability was tested at different temperatures (4 °C and 25 °C) and under light/no light exposure to simulate possible food storage conditions.

**Figure 3:**
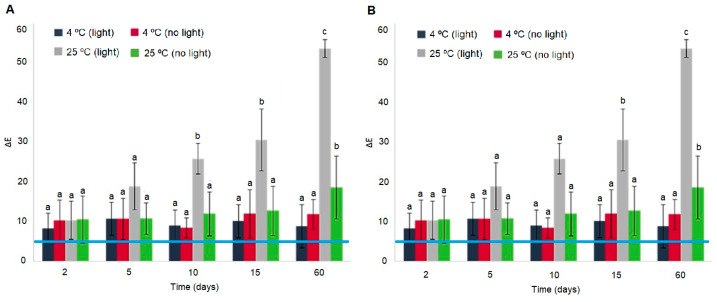
Total color difference (∆*E*) of (**A**) the BLE and (**B**) BEE films during 60 days of storage under different temperature and light conditions. Values are given as the mean ± SD (*n* = 9). The blue line represents a ∆*E* equal to 5, a value at which the consumer can visually detect colorimetric changes. ^a–c^ Different letters at the same storage condition over time represent statistically significant differences (*p* < 0.05).

As shown in Figure 3, the ∆*E* values of the BLE and BEE films were influenced by the incidence of light and temperature after 2 days of storage. The untrained human eye detects a color change when Δ*E* values are higher than 5 [33,34]. In all of the tested conditions, the BLE and BEE films presented Δ*E* values higher than 5 (Figure 3), suggesting that the films’ color change can be detected by the consumer [35]. These changes may be the result of the photodegradation of natural pigments (such as anthocyanins and betalains) in the films. However, the Δ*E* values of the BLE and BEE films remained stable from day 2 until day 60 (*p* > 0.05) when they were stored at 4 °C under the presence or absence of light. It can be concluded that if the BLE and BEE films are stored under refrigeration conditions, the films color changes will not be directly associated with the photodegradation due to light exposure, but rather to changes to the food product quality. This result is in accordance to Etxabide et al. [36], who studied the color stability of anthocyanins and betalains containing colorants under different storage conditions for the development of intelligent packaging. They concluded that the color remained stable under 4 °C and no light incidence storage conditions for 28 days, although a small color fading was observed during the final days. On the other hand, the Δ*E* results related to storage at 25 °C under light storage conditions showed an evident increase in Δ*E* values (*p* < 0.05), possibly due to BLE and BEE degradation (Figure 3). However, the BLE and BEE films showed no significant differences (*p* > 0.05) in Δ*E* values after 60 days of storage at 25 °C under no light conditions. Thus, an increase in temperature and exposure to light (e.g., during food products transport chain), which could compromise the food quality, could be detected by both films studied. Gao et al. [37] also concluded that the ∆*E* of intelligent films based on Car incorporated with anthocyanins and betalains increased gradually with the extension of storage time at 25 °C. The authors stated that the color stability decreased progressively, which may be related to the oxidation and decomposition of anthocyanins and betalains. The results of film stability as an intelligent indicator for monitoring hake spoilage during specific storage conditions were very promising. However, it should be explored in more detail in future work.

#### 3.2.3. pH-Sensitive Properties of the BLE and BEE Films

**Figure 4:**
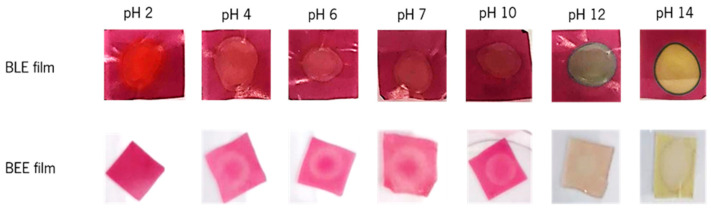
Color changes of the BLE and BEE films as a result of the contact of different buffer solutions (pH 2 to 14) to the films.

Figure 4 illustrates the visible color response (ranging from red to green color) of each film sample when exposed to different buffer solutions (pH 2 to 14). The BLE film presented a red color at pH = 2; a light purple color at a pH range of 4 to 7; a dark purple color at pH = 10; a blue/green color at pH = 12, and yellow color at pH = 14 [5,12,38]. The color parameter analysis of the BLE films showed that the L* value increased (39.13 ± 4.04 to 44.63 ± 5.87), and the a* (40.61 ± 3.02 to 26.00 ± 2.23) and b* (26.27 ± 4.16 to 10.36 ± 1.32) values significantly decreased (*p* < 0.05) between pH 2 and 4. Therefore, in acidic solutions, the BLE film showed a decrease in its red color and an increase in its blue color. The values of the L*, a*, and b* parameters remained similar (*p* > 0.05) between pH 4 and 10, which showed that the BLE film did not change its color under this pH range. As the pH value increased from 10 to 12, the L* values increased (45.05 ± 2.29 to 51.05 ± 3.35), and the a* (28.66 ± 2.21 to 7.60 ± 5.81) and b* (9.66 ± 1.99 to 3.85 ± 2.39) values dropped sharply. Thus, it can be concluded that the BLE film showed a more intense blue color, as can be seen in Figure 4. During the pH transition from 12 to 14, the L* value increased (51.05 ± 3.35 to 62.18 ± 5.03), the a* value decreased (7.60 ± 5.81 to 5.03 ± 1.54), and the b* value increased (3.85 ± 2.39 to 26.98 ± 3.68). These results demonstrate that the film became more yellow. Research has shown that the color stability of the anthocyanins present in blueberry is influenced by external conditions such as pH, storage temperature, and incidence of light [7]. Indeed, anthocyanins are more stable in acidic than in alkaline solutions. The flavylium cation form of anthocyanins is stable under acidic solutions, with its double bonds prone to conjugation (e.g., cation hydration), forming the carbinol basic form (pink-light violet color). At pH 10, anthocyanins are in the quinoidal base form due to cation deprotonation, leading to a color change to violet. At pH 12, a color change to blue-green is observed, since the quinoidal anhydro base form occurs by deprotonation from the oxygen and carbon ring. Finally, the retro-chalcone formation occurs at pH 14 through carbinol tautomerization, which is responsible for the appearance of a yellow-green color [39]. Ma et al. [40] reported similar color changes of polyvinyl alcohol-chitosan films containing anthocyanins from mulberry, while Choi et al. [12] observed color changes of agar/potato starch films incorporating anthocyanin extracts (from purple sweet potato) from bright red at pH 2, to pink at pH 3–4, to brown at pH 5–6, and finally to greenish-brown at pH 9–10.

Regarding the BEE films, they exhibited a pink-purple color when the films were in contact with buffer solutions ranging from pH 2 to 10 (Figure 4). At this pH range, the L*, a*, and b* values did not present statistically significant differences (*p* > 0.05), concluding that the BEE film did not present visible colorimetric changes under these conditions. When the pH values changed from 10 to 12, the L* value increased (55.08 ± 1.51 to 60.17 ± 6.27), and the a* (44.55 ± 3.37 to 21.97 ± 3.54) and b* (4.94 ± 1.23 to 3.37 ± 1.72) values decreased, which means that the BEE film had a higher lightness and showed a green-blue color. At pH 12 to 14, the color film changed to yellow. In this case, the L* value (60.17 ± 6.27 to 74.50 ± 2.22) continued to increase, the a* decreased significantly (21.97 ± 3.54 to 1.07 ± 3.26), and b* increased significantly (*p* < 0.05) (3.37 ± 1.72 to 23.06 ± 1.94). Beetroot contains two groups of betalain pigments: red-violet betacyanins (at pH 4 to 10) and yellow betaxanthins (at pH > 10). Betalains are relatively stable at a pH range of 2 to 10, unlike anthocyanins [28]. For this reason, it is not easy to visually distinguish the film color change over a wide range of pH values, with the most visible change between pH 10 and 12. Similar to our results, Wu et al. [13] described the color changes of LGB/polyvinyl alcohol films incorporating betacyanins (from cockscomb flower) at different pH. The films exhibited stable reddish-purple colors at pH 3 to 8 and turned light brown or light purple at pH 9 to 12. These color changes were related to the structural degradation of betacyanins in alkaline solutions. Thus, it can be concluded that the color of all films tested were strongly pH-dependent, showing their potential to be applied in the intelligent packaging field [5,28].

#### 3.2.4. Films Response to Ammonia Vapor

Generally, the spoilage of many protein-rich animal foods can produce a high amount of volatile nitrogenous compounds (e.g., ammonia, dimethylamine, and trimethylamine) due to the deterioration of proteins, which directly affect the food products’ pH status [9]. Thus, all films were exposed to an ammonia solution to simulate the volatile nitrogenous compounds released from food products presenting a high protein content, and the colorimetric film response was evaluated.

As shown in Table 2, the control film’s color did not change over time, and the a* and b* values remained nearly constant (*p* > 0.05). As expected, the BLE film showed a significant color change from pink/violet to blue after 10 min, and pale green/yellow after 60 min when exposed to ammonia vapor. Accordingly, the a* and b* values significantly decreased (*p* < 0.05) after 30 min of exposure time, which means that the BLE film became blue/green. After 30 min, the blue/green color of the film started to fade, and some grey color portions began to appear (Table 2). The a* and b* values increased, which consequently led to a decrease in the green and blue color. After 60 min, there was a decrease in the a* and b* values again, but at 120 min, they increased again, causing another considerable color change in the film (blue to pale green/yellow). The mechanism that possibly explains these color changes is the fact that ammonia can diffuse through the films, combining with water molecules and subsequently hydrolyzing into hydroxyl ions, producing an alkaline environment in films. As previously discussed, BLE films, as exposed to increasingly alkaline conditions, change their color from pink/violet to blue, and finally, to green/yellow [41]. Jiang et al. [33] also reported that the film color from carboxymethyl-cellulose/starch/purple sweet potato anthocyanins changed from red to green after 60 min of exposure time to ammonia. Other research has shown a significant color change from reddish/pink to pale green of methylcellulose/chitosan nanofiber/barberry anthocyanin films after 15 min, and to yellow after 30 min when exposed to ammonia vapor [42].

Regarding the BEE film, the colorimetric changes were not visually significant (*p* > 0.05), as shown in Table 2. The films showed a pink/violet color during 120 min when exposed to ammonia. Overall, the a* value decreased and the b* value increased during the exposure time, which allowed for an increase in the green and yellow color of the BEE film, despite not being easily visible. The ammonia diffusion on the film surface caused alkalinization of the environment, which led to the degradation of betacyanins, changing the red color into colorless cyclo-dopa-5-O-(malonyl)-β-gucoside and yellow betalamic acid, consequently inducing minor colorimetric changes in the film [23,43]. Naghdi et al. [44] developed a starch film containing paper flower betacyanin and reported the film’s sensitivity to ammonia gas as the color changed from pink to yellow after 30 min of exposure. Qin et al. [23] observed that the pink color of the polyvinyl alcohol film/starch with red pitaya betalains faded after 10 min of exposure, and the yellowness degree gradually intensified after 20 min. Despite these results being obtained by other researchers, the BEE film developed did not significantly display color changes compared to the BLE film. This can be due to several factors: the amount of betalains incorporated in the film, the polymeric base used in the bio-based film development, and the time of exposure to ammonia or the ammonia solution concentration used.

The real-time, immediate, and sensitive reactions of colorimetric films are important properties of the color and pH-sensing intelligent packaging applications. As a result, the BLE film showed the most evident colorimetric differences in a short time of exposure to ammonia compared to the BEE film. Thus, BLE could be applied to protein-rich animal food to monitor its freshness.

#### 3.2.5. Water Contact Angle (WCA), Moisture Content (MC), Water Solubility (WS), and Water Vapor Permeability (WVP)

The films’ hydrophobicity was assessed through the measurement of the contact angle of water drop on the surface of the films. All WCAs measured were less than 90 °C, which indicated that the surface of all films had a hydrophilic character [37,45]. This result could be related to the presence of free hydrophilic groups on the surface of the LBG/Car film, which make the film more hydrophilic [37]. Moreover, no significant differences were observed between the control film (34.50 ± 5.51°) and the other films (i.e., BLE (41.43 ± 3.32°) and BEE (30.62 ± 3.62°) film samples (*p* > 0.05)). However, the BLE film presented a higher hydrophobicity (i.e., higher WCA value) than the BEE film (*p* < 0.05). Wu et al. [46] reported similar results and concluded that the addition of polyphenols (such as anthocyanins) into polysaccharide-based films led to the formation of hydrogen bonds and non-covalent hydrophobic interactions. Luchese et al. [47] also described that adding blueberry residue to starch films increased the WCA, and consequently, the film hydrophobicity.

As shown in Table 3, the MC values of the BLE and BEE films were not significantly different from the MC values of the control film (*p* > 0.05). These results were different from the ones reported by Zhai et al. [48]. The authors showed a significant decrease in the MC of the starch/polyvinyl alcohol–roselle anthocyanins films, which was attributed to the interactions between the film matrix and anthocyanins that decreased the availability of hydroxyl groups in the matrix to interact with the water molecules. The MC of the BEE films were lower than the MC of the BLE films (*p* < 0.05) (Table 3). This is possibly because less hydroxyl group interactions between the water molecules and BEE films occurred compared to BLE [5,49]. Similarly, Jamróz et al. [17] verified that the MC significantly decreased in furcellaran films by adding BEE.

The film solubilization starts with water access into the biopolymeric matrix, followed by a breakdown of hydrogen bonds and Van der Waals forces between the chains of biopolymers [50]. The WS results are shown in Table 3, and it is possible to observe that the control and BEE films lost their integrity after 8 h. This may be due to the high number of hydrophilic groups, which can form strong hydrogen bonds with water molecules, leaving the films very hydrated. On the other hand, the incorporation of BLE on the film led to a significant reduction in the WS values obtained (*p* < 0.05) compared to the other films (Table 3). This could possibly be explained by hydrophobic groups present in BLE, which decreased the film’s ability to form bonds with water molecules. Thus, interaction between the phenolic compounds in BLE with the hydroxyl groups of the LBG and Car matrix led to a more compact inner-structure formation, with stronger bonds. Huang et al. [27] reported a similar WS behavior of an agar-based film incorporating *Arnebia euchroma* root extracts rich in anthocyanins, concluding that the WS was reduced significantly due to the addition of water-insoluble extracts.

The WVP is an important feature in food packaging, which assesses the water barrier properties of the film. Many films used in the food industry must have reduced low WVP values to delay food deterioration and to extend its shelf life and ensure its quality [44]. Furthermore, it has been described that the thickness, film matrix integrity, and interactions between the functional groups of film components can influence the films’ WVP [23,44]. The results obtained for the control, BLE, and BEE films are shown in Table 3. The addition of BLE to the biopolymer-based matrix did not cause a significant effect on the WVP values of the films (*p* > 0.05). As previously discussed in the WCA, WS, and MC results section, the BLE can possibly form intermolecular interactions with LBG and Car, which limited the interactions between water vapor and film matrix [49]. Gao et al. [37] also reported that the addition of an anthocyanin-rich purple sweet potato extract had no significant effect on the WVP of the Car-based film. On the other hand, in our study, a significant increase in the WVP values were observed (*p* < 0.05) when the BEE was incorporated into the LBG/Car film matrix. It is possible that the increase in the WVP can be attributed to the BEE hydrophilicity (in agreement with WCA, WS, and MC results) and the increase in micro-paths in the network microstructure, which facilitated the diffusion of water molecules through the matrix. Zamudio-Flores et al. [51] determined that the addition of a beet extract significantly increased the WVP of oxidized starch films, which was also attributed to the hydrophilic property of the betalains present in the extract composition.

#### 3.2.6. Mechanical Properties

The intelligent film must have enough strength to withstand the mechanical stress to suit its application and retain its integrity during transport and storage [21].

The effect of the incorporation of the extracts on the mechanical properties (TS and EB) of the biopolymeric matrix are shown in Table 3. The incorporation of the BLE and BEE extracts into film significantly increased the TS and EB values compared with the control film (*p* < 0.05). This result may be related to the reduction in intermolecular interactions between the LBG and Car, improving the chain mobility and the overall film elasticity. Additionally, the observed increase in TS and EB values may be associated with the interactions between the phenolic compounds and LBG/Car groups. Accordingly, the internal structure of the film matrix with the addition of natural extracts changed, becoming more compact and able to withstand higher mechanical forces [17,37]. Zhang et al. [52] showed a significant increase in the TS and EB values in starch/polyvinyl alcohol incorporating anthocyanin-rich purple potato. Qin et al. [23] found a similar trend when betalains from red pitaya peel were incorporated in a starch-polyvinyl alcohol matrix. The authors stated that the increase in the film mechanical strength film was due to a weakening of intermolecular forces between adjacent macromolecules, which facilitated the motion of polymer chains.

### 3.3. Monitoring Hake Fish Freshness Using the Developed Colorimetric Films

#### 3.3.1. Selection of Colorimetric Film to Be Applied in Fish Freshness Study

**Figure 5:**
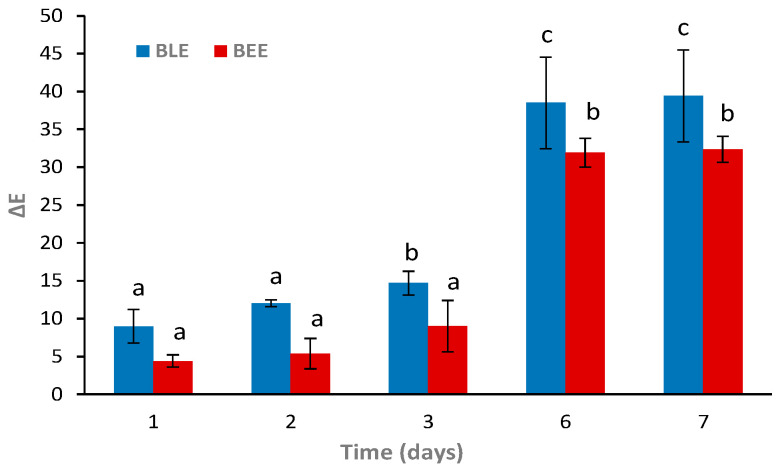
Color changes (∆E) of the BLE and BEE films during fish storage during 7 days at 4 °C. Values are given as the mean ± SD (*n* = 3). ^a–c^ Different letters indicate significant differences between the same film sample over time (*p* < 0.05).

The BLE and BEE films were tested as freshness indicators during hake storage at 4 °C. Overall, it was possible to observe gradual colorimetric changes of both films at 4 °C (Figure 5). According to previous reports, a ∆*E* value higher than 5 can be detected by the human eye. However, only values above 12 represent an absolute difference in color, which is very noticeable, even by untrained panelists/consumers [27,30]. The BLE film showed more distinguishable color variations to the naked eye (i.e., ∆*E* > 12) on day 2 of storage when compared to the BEE film (∆*E* around 5.4). At day 6, a significant increase in ∆*E* values (*p* < 0.05) occurred in both films, with colorimetric changes easily detectable. Similarly, Zhai et al. [48] reported that films based on starch/polyvinyl alcohol incorporated with roselle anthocyanins changed their color from the initial pink to purple in 3–4 days and to green after 6 days of fish storage at 4 °C. Qin et al. [23] also described that the color of starch/polyvinyl alcohol films incorporating betalains from red pitaya changed from purple to yellow after fish storage at 4 °C for 8 days.

Both colorimetric films displayed continuous color changes within the fish shelf life, suggesting that they are capable of indicating the real-time fish freshness [48]. However, the BLE film color change was more easily detectable early on (day 1) than the BEE film (day 2). Moreover, it was possible to observe a significant perceptible color change (*p* < 0.05) of the BLE film on day 3. These results could be due to the anthocyanin-rich BLE’s ability to detect more easily and react faster with volatile compounds than betalain-rich BEE. Thus, the BLE film would be more suitable for monitoring real-time fish freshness in the early days of storage than the BEE film. Hence, we decided to only apply the BLE film as an intelligent freshness indicator for the hake samples to evaluate other parameters (i.e., microbiological, pH, and TVB-N) during storage.

#### 3.3.2. Relationship between Color Changes of Freshness Indicator Film and Microbiological and Physico-Chemical Characteristics of Fish during Storage

Microbiological spoilage is one of the main factors in which to monitor fish quality during cold storage since seafood is very perishable and susceptible to microbial deterioration. This is mainly due to its higher water activity (fundamental factor for the growth and survival of microorganisms), high fat content (favorable to oxidation), and neutral pH values (useful to the development and growth of most microorganisms) when compared to other food products [30,44].

**Figure 6:**
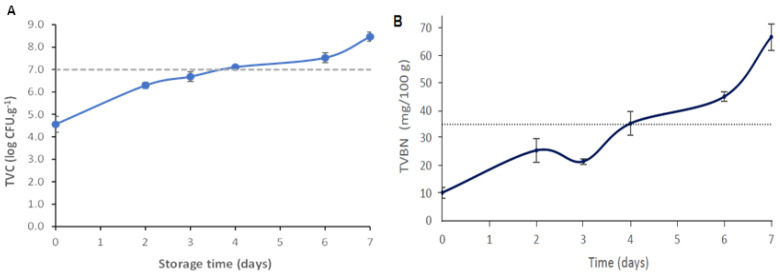
(**A**) Total viable count (TVC) of the hake samples stored for 7 days at 4 °C. The dashed horizontal line represents the maximum acceptable intake limit value (7 log CFU g^−1^. Values are the average ± SD (*n* = 6). (**B**) TVB-N levels of the hake samples during storage at 4 °C. The acceptable TVB-N limit for fish consumption (35 mg TVB-N/100 g) is represented by the dashed horizontal line. Values are the average ± SD (*n* = 3).

Therefore, the microbiological quality of the hake samples was assessed as a function of storage time. During hake storage, the microbial load increased (*p* < 0.05), being equal to 8.61 ± 0.21 log CFU g^−1^ on the last day of storage (day 7) (Figure 6A). As the storage time increases, microorganisms generally grow faster and generate various metabolites responsible for off-odors, off-flavors, texture, and color changes, resulting in sensorial rejection by the consumer [44,52]. Fish is considered tobe acceptable for human consumption if the samples’ microbial load does not exceed 7.0 log CFU g^−1^, the maximum accepted value for marine species [53]. As can be observed in Figure 6A, this limit was exceeded between days 3 and 4 of storage at 4 °C, pointing out that the hake samples had lost their quality/freshness and were no longer suitable for consumption. Huang et al. [27] described a novel colorimetric indicator based on agar incorporated with anthocyanin extracts for monitoring fish freshness at 4 °C. They concluded that the fish samples were not suitable for consumption (7.11 log CFU g^−1^) after 5–6 days.

Volatile amines such as ammonia, di-, and trimethyl amine, collectively recognized as TVB-N, are produced due to fish proteolysis during storage. For this reason, it is considered an important parameter indicative of muscle tissue degradation and the degree of fish freshness [30,44]. Usually, microbiological spoilage leads to an increase in TVB-N content because numerous enzymes produced by microorganism in the fish post-mortem phase will degrade the protein present in fish muscle tissue, and consequently, an unpleasant fish taste and odor will develop [5]. The average TVB-N values obtained throughout the storage of the hake fish samples at 4 °C are shown in Figure 6B. The TVB-N content increased significantly throughout the storage period (*p* < 0.05). On the last day of storage, the TVB-N values reached 66.78 ± 4.81 mg/100 g. This result agrees with the increase in the microbial growth on the fish samples (Figure 6A), which is related to the microbial activity and action of endogenous enzymes [23,27,30,44]. According to Regulation (EU) No. 2019/627, the TVB-N limit value for species belonging to the *Merlucciidae* family to be considered fit for human consumption is 35 mg/100 g [54]. Thus, hake samples exceeded the maximum TVB-N limit of acceptability after 4 days of storage (Figure 6B). Furthermore, it was also noticed that the lapsed time to reach the maximum TVB-N limit of acceptability (day 4) (Figure 6B) and TVC threshold (between day 3 and 4) (Figure 6A) was very similar. This phenomenon has previously been observed by other researchers who stated that the generation of TVB-N inherently follows the increase in microbial population [27].

One of the parameters associated with fish freshness and quality is the pH value. The initial pH value of the fresh hake was 6.60 ± 0.04, which gradually increased to 8.02 ± 0.03, on day 7 (Table 4). The pH increase may be correlated to compounds (such as ammonia and amines) generated from protein degradation in hake muscle, resulting from endogenous and microbial proteolytic enzyme activity [30,37,52]. The hake samples presented a higher pH value on day 4 compared to previous days (*p* < 0.05) (Table 4). The obtained pH result showed a good connection with the microbiological analyses and TVB-N results, since the threshold of the recommended TVC and TVB-N limits for fish freshness were also reached at day 4 of storage (Figure 6).

Regarding the BLE film colorimetric performance, the results showed remarkable color changes during the storage period (Table 4). The film color change from pink/purple (days 1 to 3) to blue/green (day 7) may be due to the pH sensitive characteristic of BLE films. No significant differences in the film brightness (L*) values were observed (*p* > 0.05) during storage (Table 4). It was also possible to observe that the a* value decreased significantly (*p* < 0.05) over 7 days, indicating a considerable loss of the film’s red color. It is possible that an increase in TVB-N released from the hake samples led to an increase in pH values, resulting in film color changes. According to the Table 4 results, the color of the film changed from pink to light purple from 0 to 4 days of storage, with the a* values significantly decreasing (*p* < 0.05) during this time period. Meanwhile, the TVB-N levels of hake increased from 10 to 36 mg/100 g, suggesting that the hake was spoiled and not safe for consumption (Figure 6B). Thus, it was possible to observe a correlation between the changes in the indicator film color and changes in TVB-N in hake. The reduction in hake freshness due to the microbial growth in muscle tissue (i.e., TVC increased) led to the release of increasing amounts of TVB-N (such as ammonia, trimethylamine, and dimethylacetamide) [50]. It is possible that these basic compounds were detected by anthocyanins present in BLE, leading to changes to the anthocyanins’ chemical structure (i.e., flavylium cation converted into quinoidal bases), and consequently decreasing the a* values of the films. Regarding the b* parameter, there was no significant differences (*p* > 0.05) during storage (Table 4). Similarly, Qin et al. [5] also described a color variation from purple to green/yellow of cassava starch with anthocyanins, in accordance with the change in pH and TVB-N values of pork during storage. Furthermore, a starch/polyvinyl alcohol film incorporated with roselle anthocyanins that changed color from pink/purple to green when in contact with spoiled fish has been reported [48]. The most noticeable color change for the human eye to recognize occurred on day 4, corresponding to the period when the fish was considered to be unsuitable for consumption (Table 4).

Although intelligent bio-based films have been developed in recent years for monitoring fish freshness, to the best of our knowledge, hake freshness monitoring films that are fully bio-based have not been developed yet. Moreover, studies related to the effects of light and temperature on the stability of film indicators, especially those developed with BLE, are limited. Hence, we report in this work an alternative and stable intelligent film for hake pH monitoring based on Car, LBG, and BLE, which has been successfully applied as a natural pH indicator.

The BLE-based film indicator can be integrated into packaging as a sticky label fixed to the headspace of a transparent package to monitor seafood freshness. For instance, a disc of BLE-based film with a printed ring presenting a reference color scale around it should be used for purposes of comparison.

## 4. Conclusions

Novel colorimetric LBG/Car-based films incorporating BLE or BEE as freshness indicators were successfully developed in the present work. The developed bio-based films demonstrated a high sensitivity to pH and TVB-N content changes with a visible transformation from a pink to red or yellow color under acidic or alkaline environments, respectively. Moreover, the color stability test showed that the colorimetric films were stable within 60 days at 4 °C. Based on the physicochemical properties of the developed films, the BLE film was chosen in order to evaluate its ability to monitor the hake quality during storage. The mechanical properties of the control films were enhanced when BLE was incorporated into the films as the EB and TS of the films increased 58% and 29%, respectively. Moreover, the results showed that films containing BLE became more hydrophobic (WCA = 41.43 ± 3.32°) than the control film (WCA = 34.50 ± 5.51°), which could be advantageous since water is an accelerating agent in the food deterioration process. The hake application trial suggests that the visible color transition point of the colorimetric BLE film is correlated with the loss of fish freshness due to the spoilage process over the storage time as verified by the TVC, TVB-N, and pH results. The BLE film color changed from pink to light purple after 4 days of storage, which could be observed with the naked eye, showing the shift in hake from fresh to spoiled.

In summary, this work reveals the great potential of LBG/Car-based films incorporating BLE as a dynamic colorimetric indicator suitable for sensing the actual condition of packaged and refrigerated hake, which might to some extent contribute to a reduction in food waste. Future research on the industrial use of the developed film freshness indicator including scale-up processing methods and validation tests under different storage conditions to avoid any false negative should be conducted. Furthermore, assessment of the film’s ability to act as indicators of freshness when applied to other protein-based food products such as meat should be explored.

## Figures and Tables

**Table 1:** Color parameters (L*, a* and b*) and opacity of the control, BLE, and BEE films.

Films	L*	a*	b*	Opacity (%)
Control	89.13 ± 2.51 ^a^	0.14 ± 0.05 ^a^	5.18 ± 0.82 ^a^	13.55 ± 1.81 ^a^
BLE	41.01 ± 1.46 ^b^	40.76 ± 3.20 ^b^	4.74 ± 1.90 ^b^	37.41 ± 2.73 ^b^
BEE	56.80 ± 0.84 ^b^	51.58 ± 0.96 ^c^	−4.35 ± 0.23 ^c^	24.86 ± 1.18 ^c^

^a–c^ Different superscript letters in the same column indicate significant differences (*p* < 0.05).

**Table 2:** Color response of the control, BLE, and BEE films to ammonia vapor.

	Control Film			BLE Film			BEE Film		
Time (min)	Film Photo	a*	b*	Film Photo	a*	b*	Film Photo	a*	b*
0	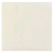	0.25 ± 0.07 ^a^	4.32 ± 0.50 ^a^	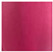	43.49 ± 3.44 ^a^	5.42 ± 2.10 ^a^	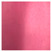	46.09 ± 4.53 ^a^	−4.96 ± 0.52 ^ab^
10	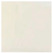	0.13 ± 0.05 ^a^	4.00 ± 0.39 ^a^	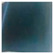	3.34 ± 2.31 ^b^	−11.59 ± 0.93 ^b^	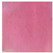	33.61 ± 3.77 ^a^	−5.70 ± 1.01 ^b^
20	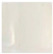	0.17 ± 0.04 ^a^	3.71 ± 0.19 ^a^	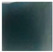	0.25 ± 0.94 ^c^	−6.35 ± 1.21 ^b^	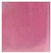	27.35 ± 4.51 ^b^	−4.45 ± 1.21 ^abc^
30	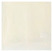	0.16 ± 0.07 ^a^	3.67 ± 0.59 ^a^	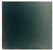	0.62 0.65 ^bc^	−3.41 ± 1.79 ^bc^	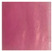	30.50 ± 3.27 ^b^	−3.83 ± 0.78 ^ab^
40	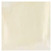	0.16 ± 0.06 ^a^	4.00 ± 0.68 ^a^	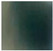	1.54 ± 1.77 ^bcd^	2.22 ± 5.38 ^bc^	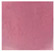	28.27 ± 4.12 ^b^	−2.24 ± 1.38 ^ad^
50	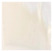	0.17 ± 0.06 ^a^	4.15 ± 0.46 ^a^	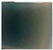	1.52 ± 1.32 ^bcd^	5.45 ± 4.33 ^c^	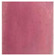	29.91 ± 2.61 ^b^	−1.82 ± 1.07 ^cd^
60	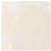	0.17 ± 0.05 ^a^	4.13 ± 0.31 ^a^	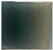	0.83 ± 1.07 ^bcd^	5.02 ± 3.88 ^c^	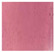	26.85 ± 7.00 ^b^	−1.18 ± 0.82 ^d^
120	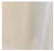	0.09 ± 0.10 ^a^	3.99 ± 0.36 ^a^	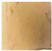	4.21 ± 1.27 ^d^	13.83 ± 4.10 ^d^	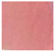	25.98 ± 5.22 ^b^	3.40 ± 2.02 ^d^

^a–d^ Different superscript letters in the same column indicate significant differences between samples (*p* < 0.05).

**Table 3:** Moisture content (MC), water solubility (WS), water vapor permeability (WVP), tensile strength (TS), and elongation-at-break (EB) of the control, BLE, and BEE films.

Films	MC (%)	WS (%)	WVP × 10^−10^ (g m^−1^ s^−1^ Pa^−1^)	TS (MPa)	EB (%)
Control	21.05 ± 1.30 ^ab^	94.41 ± 0.01 ^b^	2.09 ± 0.29 ^a^	11.70 ± 3.38 ^a^	16.50 ± 1.48 ^a^
BLE	23.54 ± 0.98 ^a^	39.66 ± 0.11 ^a^	2.30 ± 0.58 ^a^	40.26 ± 5.79 ^b^	28.39 ± 1.19 ^b^
BEE	18.23 ± 1.47 ^b^	92.90 ± 0.03 ^b^	3.13 ± 0.46 ^b^	22.83 ± 2.75 ^c^	27.76 ± 0.90 ^b^

^a–c^ Different superscript letters in the same column indicate significant differences (*p* < 0.05).

**Table 4:** pH values of hake and the color parameters (L*, a* and b*) values of the BLE film during storage at 4 °C.

Storage Time (Days)	pH	L*	a*	b*	Film Color	Freshness Stage
0	6.60 ± 0.04 ^a^	62.86 ± 3.00 ^a^	27.91 ± 1.98 ^a^	0.58 ± 0.97 ^a^	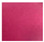	Fresh
2	6.87 ± 0.03 ^b^	50.89 ± 4.04 ^a^	20.56 ± 1.37 ^a^	3.09 ± 0.59 ^a^	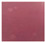	Medium fresh
3	6.68 ± 0.06 ^a^	46.47 ± 1.41 ^a^	21.59 ± 4.31 ^ab^	2.88 ± 0.55 ^a^	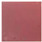	Medium fresh
4	7.04 ± 0.03 ^c^	55.41 ± 10.75 ^a^	19.25 ± 6.03 ^b^	2.69 ± 0.23 ^a^	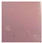	Spoiled
6	7.92 ± 0.04 ^d^	57.40 ± 9.34 ^a^	1.04 ± 0.44 ^c^	2.86 ± 2.71 ^a^	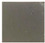	Spoiled
7	8.02 ± 0.03 ^d^	57.14 ± 5.59 ^a^	1.11 ± 0.16 ^c^	3.12 ± 2.93 ^a^	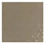	Spoiled

^a–d^ Different superscript letters in the same column indicate significant differences (*p* < 0.05).

## Data Availability

The original contributions presented in the study are included in the article, further inquiries can be directed to the corresponding author.
